# Associations between eight anthropometric indices and Parkinson’s disease: a nationwide population-based study

**DOI:** 10.3389/fnut.2025.1621658

**Published:** 2025-06-27

**Authors:** Wenting Hu, Ying Zhang, Huanxian Liu

**Affiliations:** ^1^Department of Neurology, Chengdu Integrated TCM and Western Medicine Hospital, Chengdu, China; ^2^Department of Anus and Intestine Surgery, Chengdu Integrated TCM and Western Medicine Hospital, Chengdu, China; ^3^Department of Neurology, Medical School of Chinese PLA General Hospital, Beijing, China; ^4^Yiyang Retired Cadres Sanatorium of the Hunan Province, Yiyang, China

**Keywords:** anthropometric indices, Parkinson’s disease, cross-sectional study, central obesity, NHANES

## Abstract

**Background:**

Previous studies have explored the associations between obesity and Parkinson’s disease (PD), often using body mass index (BMI) as the main metric. However, findings remain inconsistent. Anthropometric indices—quantitative measures of body shape, size, and fat distribution—offer alternative ways to assess adiposity. This study aimed to evaluate the associations between eight anthropometric indices and PD prevalence.

**Methods:**

Data were obtained from the National Health and Nutrition Examination Survey (NHANES), conducted in the U.S. from 1999 to 2020. A total of 41,374 participants aged 20 years and older were included, among whom 354 were diagnosed with PD. Eight anthropometric indices were analyzed: waist-to-weight index (WWI), conicity index (CI), a body shape index (ABSI), body roundness index (BRI), waist-to-height ratio (WHtR), BMI, waist circumference (WC), and weight (WT). Weighted multivariable logistic regression models were used to assess the association between these indices and PD. Restricted cubic spline (RCS) models were employed to examine dose–response relationships. Subgroup and sensitivity analyses were conducted to validate the robustness of the findings.

**Results:**

Significant differences were observed between the study groups, with positive and independent correlations identified between PD and all anthropometric measures, except BMI. After full adjustment, each 1-standard deviation increase in WWI, CI, ABSI, BRI, WHtR, WC, and WT was associated with an elevated PD risk by 34, 42, 36, 18, 21, 25, and 16%, respectively. RCS analysis revealed a linear relationship between CI, ABSI, BRI, WtHR, WC, WT, and PD prevalence, whereas WWI exhibited a nonlinear association. The subgroup and sensitivity analyses confirmed the consistency of these associations.

**Conclusion:**

Higher values of several anthropometric indices, particularly the ABSI, WWI, and CI, were associated with increased PD prevalence. These findings highlight the potential role of fat distribution rather than overall adiposity in PD pathogenesis. Anthropometric measures may be valuable tools for early PD risk identification and targeted prevention strategies.

## 1 Introduction

Parkinson’s disease (PD) is a progressive neurodegenerative disorder characterized by motor dysfunction, primarily manifesting as resting tremors, rigidity, bradykinesia, and postural instability (1). It is currently the second most common neurodegenerative disease after Alzheimer’s disease, affecting over 11.77 million people worldwide as of 2021 (2). With increasing life expectancy and global population aging, the prevalence of PD is expected to increase, placing a substantial burden on individuals, caregivers, and healthcare systems globally (3–6). Despite advances in symptom management, no therapies currently exist that can halt or reverse the progression of PD (7, 8). Consequently, identifying modifiable risk factors is crucial for improving early detection and prevention strategies (9).

Obesity has been implicated in various chronic diseases, including neurodegenerative disorders. However, the relationship between obesity, typically assessed using body mass index (BMI), and PD risk remains controversial. Although some studies have suggested that a higher BMI may elevate the risk of PD, others have reported no significant association or even an inverse correlation (10–16). These inconsistencies may be attributed to variations in the study populations, methodologies, and specific anthropometric measures used to assess obesity. Although BMI remains widely used in clinical and epidemiological contexts, it is increasingly recognized as a crude indicator that does not distinguish fat mass from lean mass or account for fat distribution (17). Therefore, there is an urgent need for more precise anthropometric indices to better evaluate the relationship between obesity and the risk of PD.

Recently, alternative anthropometric indices, including the body roundness index (BRI) (18, 19), a body shape index (ABSI) (20, 21), conicity index (CI) (22), waist-to-height ratio (WHtR) (23), and waist-to-weight index (WWI) (24) have been proposed to improve the assessment of fat distribution. These indices have been linked to cardiovascular diseases, type 2 diabetes, and metabolic syndrome (25–27), as they account for different patterns of fat accumulation. However, evidence regarding the association between these alternative anthropometric measures and PD risk remains limited.

This study aimed to explore the relationship between eight anthropometric indices (WWI, CI, ABSI, BRI, WHtR, BMI), waist circumference (WC), and weight (WT) and the prevalence of PD using data from the National Health and Nutrition Examination Survey (NHANES) from 1999 to 2020. By investigating these relationships, this study sought to provide new insights into the role of body fat distribution in PD development, offering potential implications for early identification and prevention strategies targeting high-risk populations.

## 2 Materials and methods

### 2.1 Data source

This cross-sectional study was conducted using publicly available secondary data from the NHANES, administered by the Centers for Disease Control and Prevention (CDC) between 1999 and 2020.^1^ The NHANES employs a complex, multistage probability sampling design to obtain a nationally representative sample of the non-institutionalized U.S. population. Data were originally collected by trained CDC personnel through mobile examination centers (MECs), which included structured household interviews, standardized physical examinations, and laboratory assessments. This study adhered to the Strengthening of the Reporting of Observational Studies in Epidemiology (STROBE) guidelines.

### 2.2 Standard protocol approval, registration, and patient consent

The National Center for Health Statistics Institutional Review Board approved the NHANES protocol, and all participants provided written informed consent for data collection. As this study involved secondary data analysis, additional Institutional Review Board approval was not required. The dataset is publicly available on the NHANES official website: https://www.cdc.gov/nchs/nhanes/index.html.

### 2.3 Study design and population

**Figure 1:**
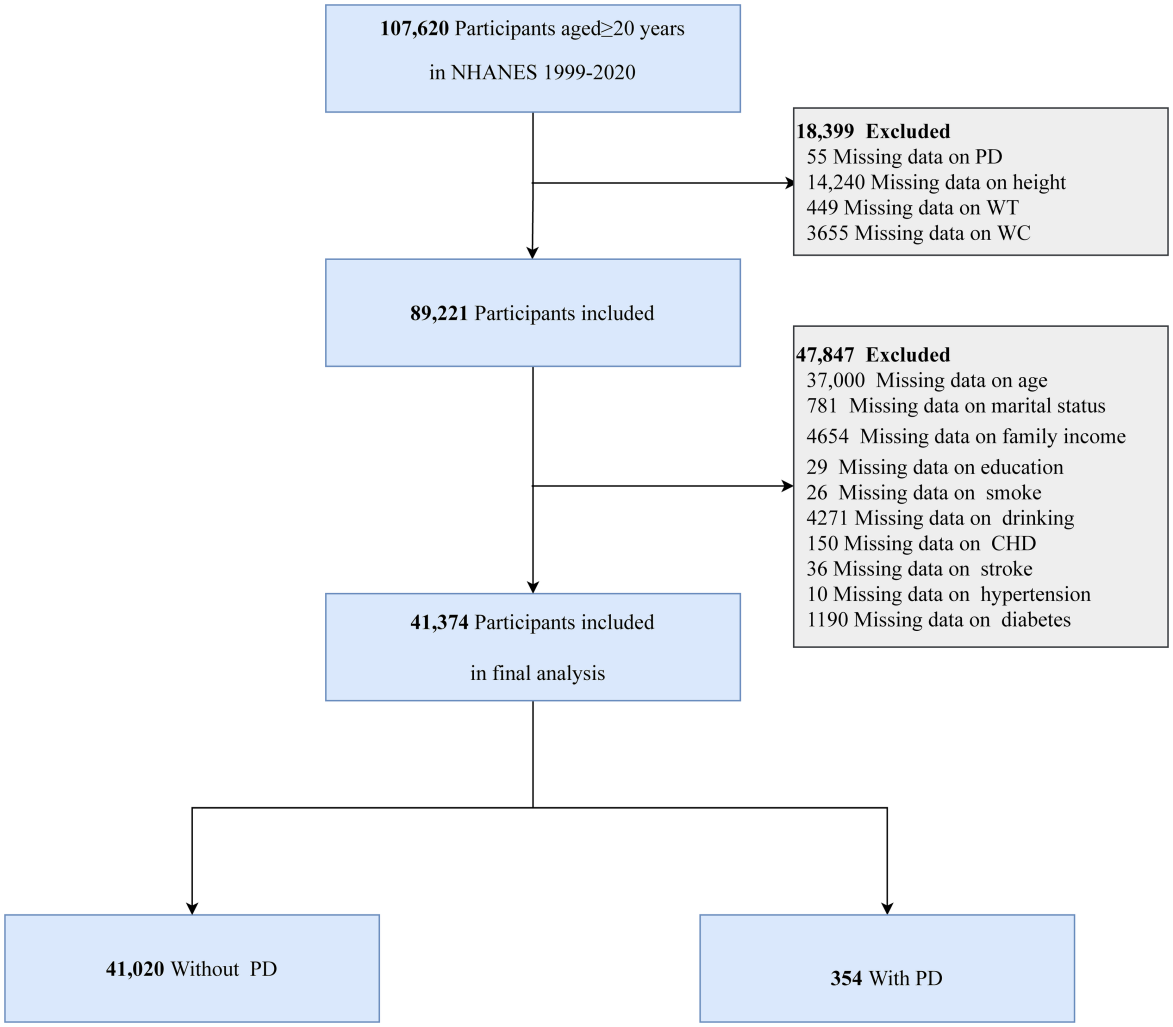
Flowchart of participants in this study.

The analysis included individuals aged 20 years and older who completed the NHANES survey and had their data available on anthropometric measurements and PD. Several exclusion criteria were applied to ensure the validity of the dataset. Participants were excluded if they had missing data on height, WT, WC, and PD, or lacked data on relevant covariates. Figure 1 illustrates the specific inclusion and exclusion criteria in detail.

### 2.4 Anthropometric index calculation

Trained examiners measured key anthropometric parameters, including height, WC, and WT, using standardized protocols at MEC. WC was measured to the nearest millimeter at the end of normal exhalation while the participant stood upright with feet 25–30 cm apart. The following anthropometric indices were calculated using established formulas (28):

BMI = WT (kg)/height^2^ (m^2^)

ABSI = WC (cm)/[BMI^2/3^ (kg/m^2^) × height^1/2^ (m)]

BRI = 364.2–365.5 × {1 − [(WC (cm)/2π)/(0.5 × height (m))]^2^}^0.5^

CI = WC (m)/0.109/[WT (kg)/height (cm)]^0.5^

WWI = WC (cm)/WT^0.5^ (cm/kg^0.5^)

WHtR = WC (cm)/height (cm)

### 2.5 Assessment of PD

PD was identified based on self-reported prescription medication records in NHANES, specifically under the category “Anti-Parkinson Agents.” Only participants actively receiving antiparkinsonian medications were classified as having PD, whereas those without relevant prescriptions were categorized as non-PD. This definition is consistent with the previously established definitions in the literature (29–31).

### 2.6 Assessment of covariates

Potential confounders were selected based on prior literature and clinical relevance (31–33). These variables were categorized into four main domains: demographic, socioeconomic, lifestyle, and medical history. Details of the specific covariates and their classification criteria are summarized in Supplementary Table 1.

Briefly, demographic variables included age, sex, and race/ethnicity. Socioeconomic factors comprised marital status, poverty-income ratio (PIR), and educational attainment. Lifestyle variables included smoking status (31), alcohol consumption, and physical activity measured in metabolic equivalent of task (MET)-minutes [PA(MET-min/wk)] per week based on previously defined activity levels (34, 35). Medical history included physician-diagnosed coronary heart disease (CHD), stroke, hypertension, hyperlipidemia, and diabetes (all classified as yes/no). Hypertension, hyperlipidemia, and diabetes were defined using standard clinical and laboratory criteria.

### 2.7 Statistical analyses

Following the NHANES analytical guidelines, survey design variables and appropriate sampling weights were applied to ensure nationally representative estimates. Sampling weights were calculated as follows: 1999–2002: 2/10.6 × 4-year MEC weight, 2003–2016: 1/10.6 × 2-year MEC weight, and 2017–2020: 1.6/10.6 × PRP MEC weight.

The baseline characteristics are presented in Table 1, with continuous variables with a normal distribution reported as mean [standard deviation (SD)] whereas, non-normally distributed variables are presented as medians with interquartile ranges (IQR). Categorical variables were expressed as unweighted numbers (weighted percentages). The Kolmogorov–Smirnov test was applied to assess the normality of the distributions. Group comparisons were performed using the chi-square test for categorical variables, independent sample *t*-tests for normally distributed continuous variables, and the Mann–Whitney U test for non-normally distributed continuous variables. Given the large sample size, missing data were handled by excluding incomplete records from the analysis. In this study, all anthropometric variables underwent z-score transformation using the formula: z-score = (index-index_mean_)/index_sd_ (Supplementary Table 2).

**Table 1:** Baseline characteristics of participants, weighted.

Characteristics	Participants^a^			
	Overall	Non-PD	PD	*p*-value^b^
	*n* = 41,374	*n* = 41,020	*n* = 354	
Age (years), mean (SD)	46.81(16.64)	46.73(16.63)	56.80(15.21)	**<0.0001**
Sex, *n* (%)				0.014
Male	20,828 (49.36%)	20,659 (49.43%)	169 (40.14%)	
Female	20,546 (50.64%)	20,361 (50.57%)	185 (59.86%)	
Race, *n* (%)				**0.011**
Non-Hispanic White	19,002 (70.28%)	18,786 (70.21%)	216 (79.09%)	
Non-Hispanic Black	8,669 (10.42%)	8,611 (10.44%)	58 (8.79%)	
Mexican American	6,816 (7.63%)	6,776 (7.66%)	40 (4.09%)	
Other Hispanic	3,299 (5.32%)	3,273 (5.33%)	26 (3.87%)	
Other	3,588 (6.35%)	3,574 (6.37%)	14 (4.15%)	
Marital status, *n* (%)				0.15
Married or living with a partner	24,991 (64.27%)	24,799 (64.32%)	192 (58.45%)	
Living alone	16,383 (35.73%)	16,221 (35.68%)	162 (41.55%)	
Family income, *n* (%)				**<0.001**
≤1.30	12,299 (20.21%)	12,168 (20.15%)	131 (28.54%)	
1.31–3.50	15,687 (35.44%)	15,551 (35.41%)	136 (40.22%)	
>3.50	13,388 (44.34%)	13,301 (44.45%)	87 (31.24%)	
Education level, *n* (%)				0.46
Less than 9th grade	4,327 (4.97%)	4,285 (4.96%)	42 (6.39%)	
9-11th grade (includes12th grade with no diploma)	5,843 (10.52%)	5,783 (10.50%)	60 (12.57%)	
High school graduate GED or equivalent	9,618 (23.95%)	9,540 (23.94%)	78 (25.45%)	
Some college or AA degree	12,182 (31.48%)	12,077 (31.48%)	105 (31.22%)	
College graduate or above	9,404 (29.09%)	9,335 (29.13%)	69 (24.37%)	
Smoking status, *n* (%)				0.10
Never	22,200 (53.59%)	22,031 (53.60%)	169 (51.74%)	
Former	10,336 (25.07%)	10,240 (25.10%)	96 (21.45%)	
Current	8,838 (21.35%)	8,749 (21.30%)	89 (26.81%)	
Drinking status, *n* (%)				**<0.001**
Never	5,578 (10.60%)	5,529 (10.60%)	49 (11.18%)	
Former	6,786 (13.33%)	6,680 (13.25%)	106 (23.95%)	
Current	29,010 (76.07%)	28,811 (76.16%)	199 (64.87%)	
PA (MET-min/wk), median (IQR)	740.01 (80.00, 2,880.00)	756.00 (80.00, 2,880.00)	360.00 (0.00, 1,680.00)	**<0.001**
Coronary heart disease, *n* (%)				**0.009**
No	39,680 (96.61%)	39,356 (96.64%)	324 (93.35%)	
Yes	1,694 (3.39%)	1,664 (3.36%)	30 (6.65%)	
Stroke, *n* (%)				**<0.001**
No	39,873 (97.34%)	39,556 (97.40%)	317 (90.39%)	
Yes	1,501 (2.66%)	1,464 (2.60%)	37 (9.61%)	
Hyperlipidemia, *n* (%)				0.056
No	12,558 (31.18%)	12,475 (31.24%)	83 (24.65%)	
Yes	28,816 (68.82%)	28,545 (68.76%)	271 (75.35%)	
Hypertension, *n* (%)				**<0.001**
No	23,897 (63.24%)	23,755 (63.39%)	142 (44.99%)	
Yes	17,477 (36.76%)	17,265 (36.61%)	212 (55.01%)	
Diabetes, *n* (%)				**<0.001**
No	34,382 (87.58%)	34,131 (87.65%)	251 (78.06%)	
Yes	6,992 (12.42%)	6,889 (12.35%)	103 (21.94%)	
BMI [kg/m^2^, mean (SD)]	28.789 (6.670)	28.782 (6.670)	29.712 (6.662)	**0.0446**
WC (cm), mean (SD)	98.537 (16.329)	98.498 (16.331)	103.372 (15.277)	**<0.001**
WT (kg), mean (SD)	82.452 (21.144)	82.444 (21.154)	83.521 (19.810)	0.5226
ABSI, mean (SD)	0.081 (0.005)	0.081 (0.005)	0.084 (0.005)	**<0.001**
BRI, mean (SD)	5.254 (2.262)	5.248 (2.261)	6.018 (2.276)	**<0.001**
WWI, mean (SD)	10.906 (0.822)	10.902 (0.821)	11.370 (0.767)	**<0.001**
WHtR, mean (SD)	0.580 (0.097)	0.584 (0.096)	0.618 (0.094)	**<0.001**
CI, mean (SD)	1.299 (0.092)	1.299 (0.092)	1.349 (0.084)	**<0.001**

a Normally distributed continuous variables are described as means (SD), and continuous variables without a normal distribution are presented as medians (interquartile ranges). Categorical variables are presented as unweighted numbers (weighted percentages).

b Wilcoxon rank-sum test for complex survey samples; chi-squared test with Rao & Scott’s second-order correction. *p*-values < 0.05 are presented in bold.

PD, Parkinson’s disease; WWI, weight-adjusted waist index; CI, conicity index; ABSI, a body shape index; BRI, body roundness index; WHtR, waist-to-height ratio; BMI, body mass index; WC, waist circumference; WT, weight.

To explore the associations between anthropometric indices and PD risk, weighted multivariable logistic regression models were constructed to estimate odds ratios (ORs) and 95% confidence intervals (CIs). The models were adjusted as follows: Model 1: unadjusted; Model 2: adjusted for age, sex, and race/ethnicity; Model 3: further adjusted for marital status, family income, educational level, smoking status, alcohol consumption, physical activity, CHD, stroke, hypertension, diabetes, and hyperlipidemia.

A weighted restricted cubic spline (RCS) model with three knots was used to assess the potential dose–response relationships. Additionally, smoothed curve fitting was applied to evaluate the linearity of the associations.

Subgroup analyses and interaction tests were conducted to determine whether associations varied across key demographic and clinical subgroups, including age (<60 vs. ≥60 years), sex (male vs. female), race (non-Hispanic White vs. other), marital status (married/living with a partner vs. living alone), and the presence of hypertension (no vs. yes), diabetes (no vs. yes), and hyperlipidemia (no vs. yes). To ensure robustness, sensitivity analyses were performed by categorizing anthropometric indices into quartiles.

All statistical analyses were performed using R 4.2.2 (http://www.Rproject.org; The R Foundation, Vienna, Austria) and Free Statistics software (version 2.1; Beijing Free Clinical Medical Technology Co., Ltd., Beijing, China). A two-sided *p*-value <0.05 was considered statistically significant. Data analyses were conducted between December 2024 and March 2025.

## 3 Results

### 3.1 Study population

The NHANES survey, conducted from 1999 to 2020, initially included 107,620 individuals aged 20 years or older. A total of 66,246 participants were excluded based on the following criteria: 55 due to missing PD data, 14,240 due to incomplete height measurements, 449 due to missing WT data, 3,655 due to unavailable WC measurements, and 47,847 due to incomplete covariate data. Thus, the final analysis included 41,374 participants (Figure 1).

### 3.2 Baseline characteristics

Table 1 presents the baseline characteristics of participants with complete data on PD and anthropometric indices. Among the 41,374 individuals analyzed, 354 (0.86%) had PD. Compared to individuals without PD, participants with PD had significantly higher values for most anthropometric indices. Additionally, the PD group demonstrated a significantly lower prevalence of alcohol consumption and regular physical activity than the non-PD group (*p* < 0.05). Moreover, the PD group had a higher mean age at disease onset and a greater proportion of females. Individuals with PD are also more likely to live alone and report their current smoking status. Notably, the prevalence of comorbid conditions, such as CHD, hypertension, stroke, hyperlipidemia, and diabetes, was significantly higher in the PD group compared to the non-PD group.

### 3.3 Associations between eight anthropometric measures and PD

Most anthropometric indices were positively correlated with the prevalence of PD (Table 2). In the unadjusted model (Model 1), WWI showed the strongest association per 1-SD increment (OR: 1.76; 95% CI: 1.56–1.99; *p* < 0.001).

**Table 2:** Weighted logistic regression analysis of anthropometric indices and PD.

Variables	Model 1		Model 2		Model 3	
	OR (95%CI)	*P*-value^a^	OR (95%CI)	*P*-value	OR (95%CI)	*P*-value
WWI Z-score	1.76 (1.56, 1.99)	**<0.001**	1.45 (1.25, 1.68)	**<0.001**	1.34 (1.13, 1.60)	**0.001**
CI Z-score	1.73 (1.53, 1.97)	**<0.001**	1.49 (1.30, 1.71)	**<0.001**	1.42 (1.23, 1.63)	**<0.001**
ABSI Z-score	1.76 (1.55, 1.99)	**<0.001**	1.47 (1.27, 1.70)	**<0.001**	1.36 (1.17, 1.58)	**<0.001**
BRI Z-score	1.33 (1.21, 1.46)	**<0.001**	1.23 (1.10, 1.37)	**<0.001**	1.18 (1.05, 1.34)	**0.006**
WHtR Z-score	1.38 (1.24, 1.53)	**<0.001**	1.25 (1.11, 1.42)	**<0.001**	1.21 (1.06, 1.38)	**0.005**
BMI Z-score	1.14 (1.01, 1.28)	**0.030**	1.14 (1.00, 1.29)	**0.048**	1.11 (0.99, 1.26)	0.078
WC Z-score	1.31 (1.17, 1.48)	**<0.001**	1.28 (1.13, 1.45)	**<0.001**	1.25 (1.12, 1.40)	**<0.001**
WT Z-score	1.05 (0.91, 1.22)	0.513	1.16 (1.01, 1.34)	**0.042**	1.16 (1.03, 1.31)	**0.015**

Model 1: unadjusted model. Model 2: adjust for gender, age, race. Model 3: adjust for age, sex, race, marital status, family income, educational level, smoking status, alcohol drinking status, physical activity, coronary heart disease, stroke, hypertension, diabetes, and hyperlipidemia. OR, odds ratio; CI, confidence interval; PD, Parkinson’s disease; WWI, weight-adjusted waist index; CI, conicity index; ABSI, a body shape index; BRI, body roundness index; WHtR, waist-to-height ratio; BMI, body mass index; WC, waist circumference; WT, weight. ^a^*p*-values < 0.05 are presented in bold.

After adjusting for potential confounders, including age, sex, race, marital status, income, education, smoking, alcohol consumption, physical activity, and comorbidities, the associations remained significant for seven anthropometric indices in Model 3: WWI (OR: 1.34; 95% CI: 1.13–1.60; *p* < 0.001), CI (OR: 1.42; 95% CI: 1.23–1.63; *p* < 0.001), ABSI (OR: 1.36; 95% CI: 1.17–1.58; *p* < 0.001), BRI (OR: 1.18; 95% CI: 1.05–1.34; *p* = 0.006), WHtR (OR: 1.21; 95% CI: 1.06–1.38; *p* = 0.005), WC (OR: 1.25; 95% CI: 1.12–1.40; *p* < 0.001), and WT (OR: 1.16; 95% CI: 1.03–1.31; *p* = 0.015).

Compared to other anthropometric indices, BMI exhibited only a weak association with PD in the unadjusted model and Model 2, and this association became non-significant after full adjustment in Model 3.

### 3.4 Dose–response relationships based on RCS

**Figure 2:**
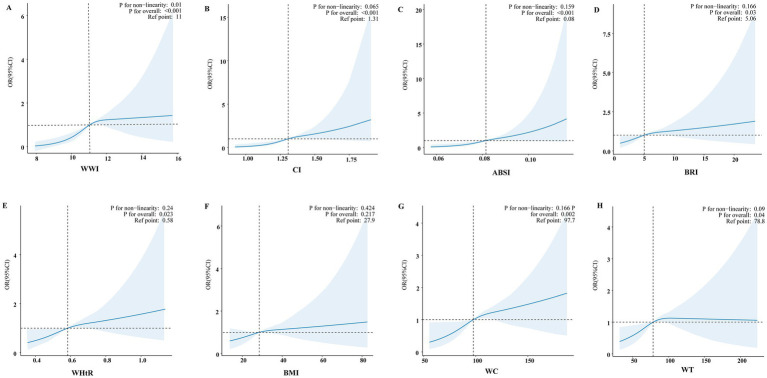
Smooth curve fitting models evaluated the correlation between eight anthropometric indices and PD. Adjusted smooth curve fitting models adjusted for age, sex, race, marital status, family income, educational level, smoking status, alcohol drinking status, physical activity, coronary heart disease, stroke, hypertension, diabetes, and hyperlipidemia. The blue line illustrates the smoothed curve that fits the data points, while the light blue shaded areas indicate the 95% CI around the fit. **(A)** Smooth curve fitting model of WWI. **(B)** Smooth curve fitting model of CI. **(C)** Smooth curve fitting model of ABSI. **(D)** Smooth curve fitting model of BRI. **(E)** Smooth curve fitting model of WHtR. **(F)** Smooth curve fitting model of BMI. **(G)** Smooth curve fitting model of WC. **(H)** Smooth curve fitting model of WT.

An additive generalized model and smoothed curve fitting were applied to examine the relationship between PD prevalence and anthropometric indicators (Figure 2). The analysis revealed a nonlinear association between WWI and PD prevalence (*P* for nonlinearity = 0.01, Figure 2A), with an inflection point identified at 11.46 cm/√kg (Table 3). In contrast, CI, ABSI, BRI, WHtR, BMI, WC, and WT exhibited a positive linear association with the prevalence of PD (*P* for nonlinearity > 0.05, Figures 2B–H).

**Table 3:** Threshold effect analysis of the relationship of WWI with PD.

WWI	Adjusted model	
	OR (95%CI)	*P*-value^a^
<11.46	1.77 (1.20, 2.61)	**0.004**
≥11.46	1.20 (0.81, 1.76)	0.360

WWI, weight-adjusted waist index; OR, odds ratio; CI, confidence intervals; PD, Parkinson’s disease. ^a^*p*-values < 0.05 are presented in bold.

### 3.5 Subgroup analyses

**Figure 3:**
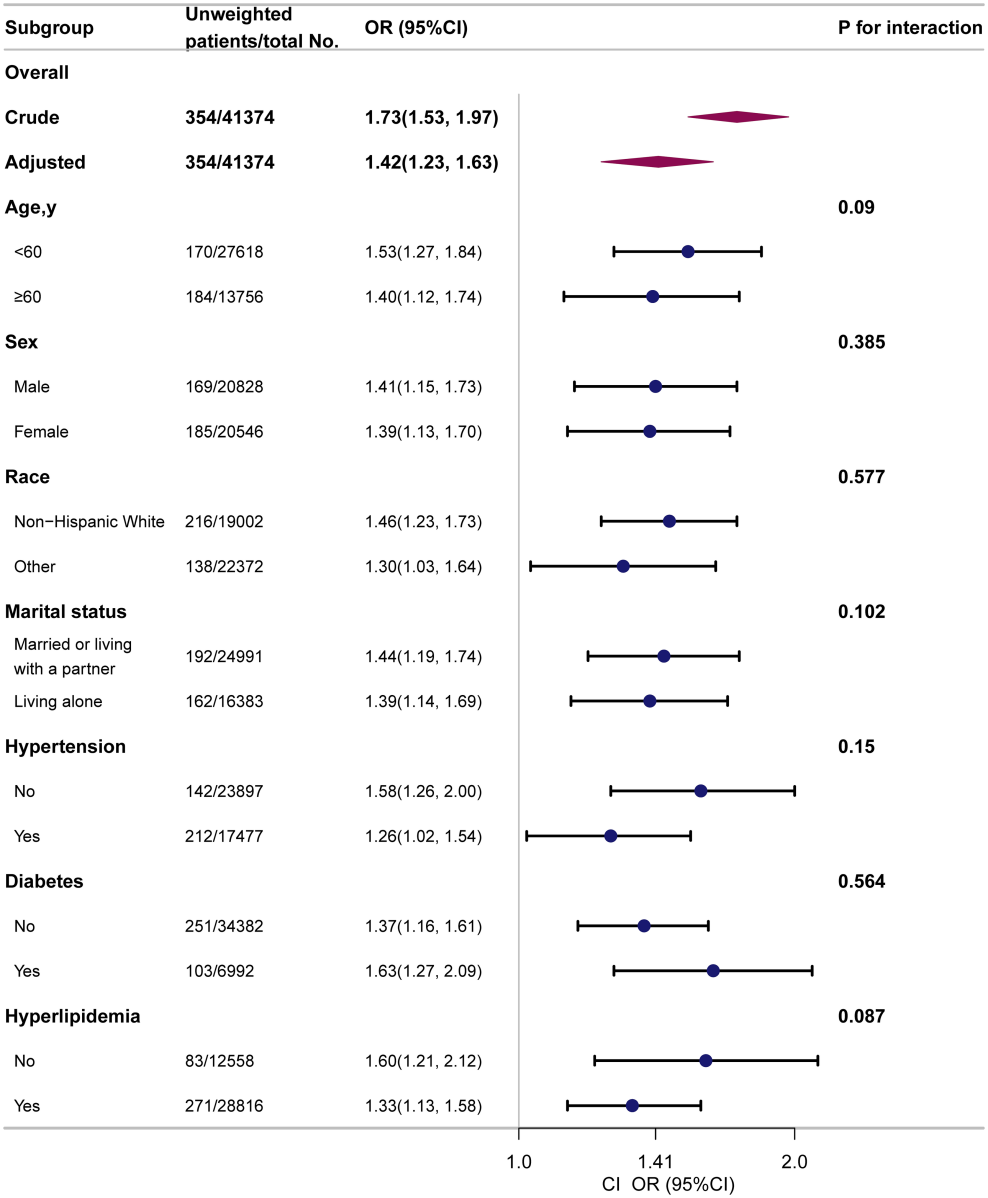
Subgroup analyses to determine the correlation of CI and PD. Except for the stratification factor itself, the stratifications were adjusted for all variables (age, sex, race, marital status, family income, educational level, smoking status, alcohol drinking status, physical activity, coronary heart disease, stroke, hypertension, diabetes, hyperlipidemia).

Subgroup analyses showed consistent positive associations between several anthropometric indices and PD prevalence. The association between CI and PD is presented in the main manuscript (Figure 3). Additional associations for WWI and ABSI are detailed in Supplementary Figure 1, for BRI and WHtR in Supplementary Figure 2, and for WC and WT in Supplementary Figure 3. Across all stratified analyses (by age, sex, race, marital status, and comorbidities), no significant interaction effects were observed.

### 3.6 Sensitivity analysis

To further assess the robustness of our findings, the anthropometric indices were categorized into quartiles. Participants in the highest quartile of WWI, CI, ABSI, BRI, WHtR, WC, and WT exhibited a significantly higher risk of PD compared to those in the lowest quartile: WWI (OR: 3.22; 95% CI: 1.77–5.85; *p* < 0.001), CI (OR: 2.74; 95% CI: 1.67–4.49; *p* < 0.001), ABSI (OR: 2.61; 95% CI: 1.53–4.43; *p* < 0.001), BRI (OR: 1.74; 95% CI: 1.05–2.88; *p* = 0.031), WHtR (OR: 1.74; 95% CI: 1.05–2.88; *p* = 0.031), WC (OR: 2.10; 95% CI: 1.31–3.37; *p* = 0.002), and WT (OR: 1.74; 95% CI: 1.14–2.66; *p* = 0.011) (Supplementary Table 3).

When BMI was analyzed as a categorical variable, no significant association with PD was observed across all models.

These findings reinforce the robustness of the observed associations and demonstrate that the associations between central obesity-related indices and PD remain consistent across different statistical modeling approaches.

## 4 Discussion

In this nationally representative study, seven of the eight anthropometric indices examined were significantly associated with a higher prevalence of PD, with BMI being the only index that did not demonstrate a significant association. These findings highlight the relevance of fat distribution rather than general adiposity in PD pathogenesis. Traditional measures, such as BMI, may underestimate neurodegenerative risk by failing to capture visceral fat accumulation, which is more closely linked to metabolic dysfunction and inflammation (36–39).

Although WC and WHtR are widely accepted indicators of central obesity, other indices evaluated in this study, including WWI, CI, ABSI, and BRI, also reflect central fat deposition. Prior research has established strong correlations between these alternative indices, visceral adiposity and both metabolic and neurological health (37, 40–43). Unlike BMI, these indices account for variations in body composition and fat distribution and have demonstrated superior predictive value in identifying individuals with central obesity. Therefore, the consistent associations observed across these measures provide further support for a shared pathophysiological mechanism linking central obesity and PD.

Our findings align with those of several previous studies that have reported a positive association between abdominal adiposity and PD risk. For instance, a large-scale cohort study in Asia involving 6.9 million individuals with an 8.35-year follow-up reported a significant association between higher WC and increased PD risk (OR = 1.09; 95% CI: 1.07–1.12; *p* < 0.001) (43). Similarly, a meta-analysis conducted by Fang et al. suggested that being overweight may contribute to increased susceptibility to PD (44). Additionally, a recent NHANES-based study demonstrated that the ABSI was positively associated with PD prevalence, particularly in younger male adults (45). Collectively, these results support the hypothesis that central obesity is a critical component in neurodegenerative risk profiling.

However, our findings differ from those of several previous studies, which reported inverse associations between BMI and PD (11, 14, 15, 16). In our analysis, although BMI showed a weak association with PD in the unadjusted and partially adjusted models, this association became non-significant after full adjustment for demographic, lifestyle, and clinical factors. This attenuation may be attributed to several reasons. First, BMI lacks specificity in distinguishing fat mass from muscle mass and fails to capture fat distribution (42, 46). In contrast, indices such as WWI, CI, and WHtR are more sensitive to the visceral fat content, which is metabolically active and implicated in neuroinflammation. Second, sample heterogeneity across the studies may contribute to the divergent outcomes. Our study included a racially and socioeconomically diverse cohort, whereas other investigations may have included more homogeneous groups. Third, survivorship bias in longitudinal cohorts may obscure the true associations, as individuals with higher BMI may die prematurely from cardiovascular diseases or diabetes before developing PD. Lastly, statistical power and variable-covariate adjustments across studies may influence the detection of associations. These factors underscore the importance of methodological rigor and index selection in studies examining adiposity and the risk of PD.

Several biological mechanisms may explain the observed associations. First, central obesity promotes systemic chronic inflammation. Adipose tissue in individuals with excess visceral fat produces proinflammatory cytokines, such as tumor necrosis factor-alpha (TNF-*α*), interleukin-6 (IL-6), and C-reactive protein (CRP). These mediators can impair the integrity of the blood–brain barrier (47–50), activate microglial cells, and promote neuroinflammation, which collectively may contribute to dopaminergic neuron degeneration in the substantia nigra, ultimately increasing PD risk (51, 52).

Second, central adiposity is closely linked to insulin resistance (IR), a metabolic state known to contribute to neurodegenerative processes. IR may lead to mitochondrial dysfunction, increase oxidative stress, and promote the aggregation of α-synuclein, a pathological hallmark of PD (51, 53–55). Moreover, IR can disrupt key intracellular signaling pathways, such as polo-like kinase 2 (PLK2), which are involved in neuronal survival and function. These alterations may accelerate the degeneration of dopaminergic neurons and exacerbate disease progression (36, 53, 56, 57). Given that central obesity is a well-established predictor of IR, it may serve as a key metabolic link between obesity and PD (58).

Third, obesity is associated with decreased levels of brain-derived neurotrophic factor (BDNF), a protein essential for neuronal development, plasticity, and survival (59, 60). Decreased levels of BDNF expression have been observed in individuals with obesity and may impair the maintenance and function of dopaminergic neurons. This deficiency in neurotrophic support further contributes to the pathology of PD (61, 62).

These biological mechanisms, including inflammation, IR, and impaired neurotrophic signaling, highlight the biological plausibility of the link between central obesity and PD. A simplified schematic is presented in Supplementary Figure 4, summarizing how central obesity may contribute to PD pathogenesis through three interconnected mechanisms.

From a clinical perspective, these findings have important clinical implications for the early detection and targeted prevention strategies. Alternative anthropometric indices could serve as practical tools for early identification of individuals at increased risk of PD, particularly in populations where BMI fails to reflect the true metabolic burden. These indices can be incorporated into routine screening protocols to inform the development of preventive strategies.

This study has several strengths. First, it was based on a large nationally representative sample, which enhanced the generalizability of the findings. Second, it incorporated standardized anthropometric measurements and employed rigorous statistical adjustments for a wide range of potential confounders. Third, the robustness of the associations was verified using subgroup and sensitivity analyses.

Despite these strengths, several limitations of this study must be acknowledged. As this was a cross-sectional study, causal relationships could not be established. To address this, future research should employ prospective cohort designs to clarify the temporal and potentially causal relationships between central obesity and PD. Second, consistent with previously published research (33, 63, 64), PD identification based on antiparkinsonian medication use may not distinguish PD from other forms of parkinsonism. This could lead to potential misclassification and underestimation of true PD prevalence, particularly among untreated individuals. Future studies should incorporate multiple diagnostic approaches, such as clinical interviews, neurological examinations, and biomarker assessments, to enhance classification accuracy. Additionally, the use of secondary data limited our ability to fully account for all confounding variables, thereby introducing the possibility of residual bias. Lastly, although the NHANES dataset is representative of the U.S. population, these findings may not be generalizable to other geographic or ethnic populations. Future validation studies in more diverse international populations are warranted to confirm and extend these results.

## 5 Conclusion

This study provided strong evidence that central obesity, as reflected by alternative anthropometric indices such as CI, WHtR, and WWI, is significantly associated with PD prevalence, whereas BMI is not. These results highlight the importance of using alternative anthropometric tools for identifying individuals at an elevated PD risk. Future longitudinal studies should explore whether interventions targeting central obesity reduce the incidence and progression of PD.

## Data Availability

The datasets presented in this study can be found in online repositories. The names of the repository/repositories and accession number(s) can be found at: https://www.cdc.gov/nchs/nhanes/index.html.

## Footnotes

^1^ https://www.cdc.gov/nchs/nhanes/about/index.html

## Glossary

NHANES: National Health and Nutrition Examination Survey
PD: Parkinson’s disease
WC: waist circumference
WT: weight
WWI: weight-adjusted waist index
CI: conicity index
ABSI: a body shape index
BRI: body roundness index
WHtR: waist-to-height ratio
BMI: body mass index
CDC: Centers for Disease Control and Prevention
MEC: mobile examination centers
STROBE: Strengthening the Reporting of Observational Studies in Epidemiology
NCHS: National Center for Health Statistics
MET: metabolic equivalent of task
CHD: coronary heart disease
IRB: Institutional Review Board
PIR: poverty income ratio
TG: triglycerides
LDL-C: low-density lipoprotein cholesterol
HDL-C: high-density lipoprotein cholesterol
SD: standard deviation
IQR: interquartile ranges
OR: odds ratio
CI: confidence interval
RCS: restricted cubic spline
TNF-*α*: tumor necrosis factor-alpha
IL-6: interleukin-6
CRP: C-reactive protein
BBB: blood–brain barrier
PLK2: Polo-Like Kinase 2
SNCA: α-synuclein
BDNF: brain-derived neurotrophic factor
