# Supplementary figures and images for: Associations between eight anthropometric indices and Parkinson’s disease: a nationwide population-based study
Source: Front Nutr. 2025 Jun 27;12:1621658. doi: 10.3389/fnut.2025.1621658 (PMC12245708; doi:10.3389/fnut.2025.1621658)

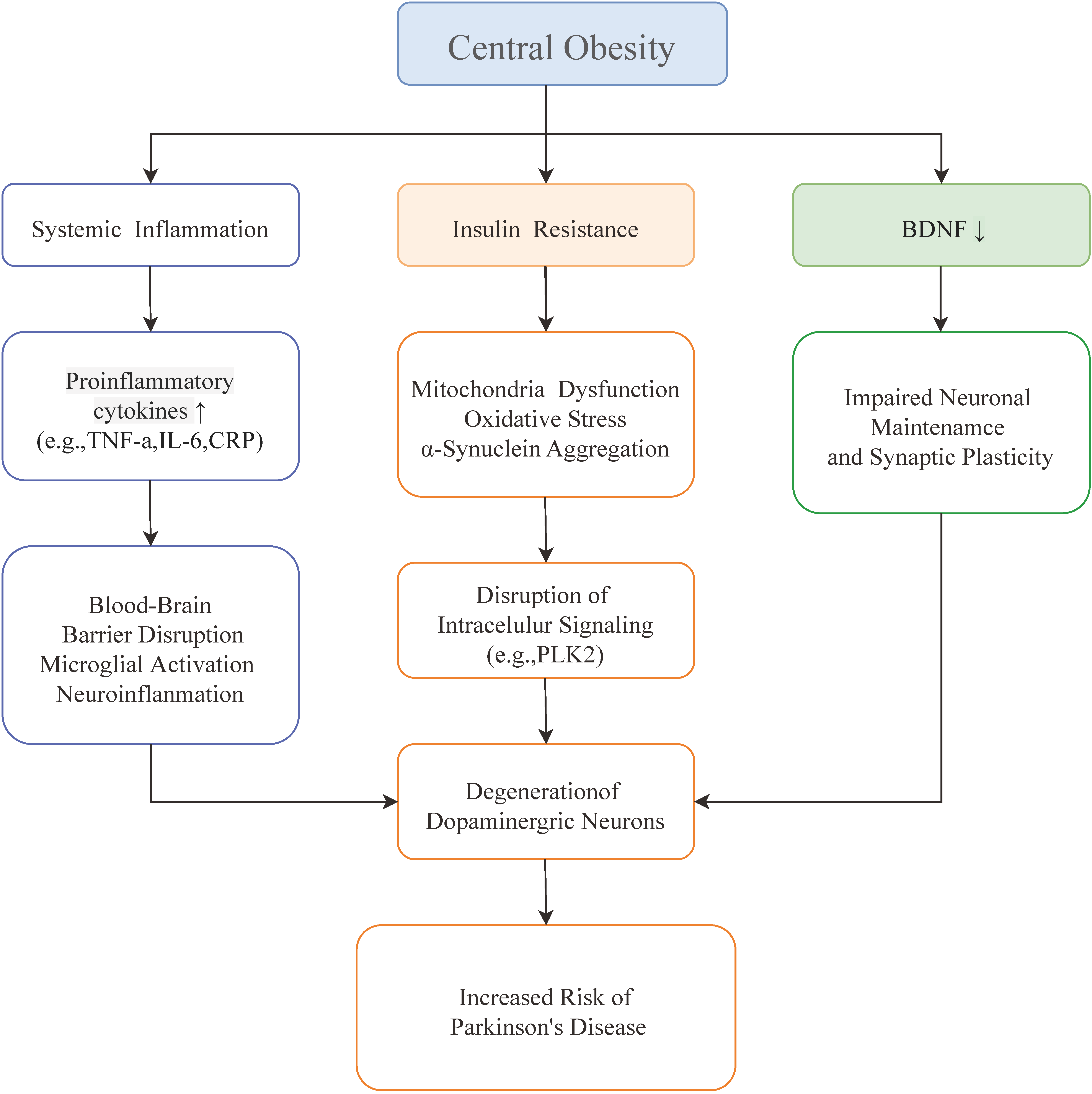

Supplement: Supplementary file 4 [file Image_4.tiff]
