# Supplementary material for: Associations between eight anthropometric indices and Parkinson’s disease: a nationwide population-based study
Source: Front Nutr. 2025 Jun 27;12:1621658. doi: 10.3389/fnut.2025.1621658 (PMC12245708; doi:10.3389/fnut.2025.1621658)
Supplement: Supplementary file 6 [file Table_1.docx]

**Supplementary Table 1** Summary of covariates included in the multivariable regression models.

| **Factor** | **Confounder** | **Description** |
| --- | --- | --- |
| Demographic | Age | Continuous variable |
|  | Sex | Male / Female |
|  | Race/Ethnicity | Non-Hispanic White, Non-Hispanic Black, Mexican American, Other Hispanic, Other |
| Socioeconomic | Marital Status | Married / Living with partner / Living alone |
|  | Family Income (PIR) | Low (<1.3), Medium (1.3–3.5), High (≥3.5) |
|  | Education Level | <9th grade, 9–11th grade, High school/GED or equivalent, Some college/AA degree, College graduate or above |
| Lifestyle | Smoking Status | Never, Former, Current |
|  | Alcohol Consumption | Never, Former, Current |
|  | Physical Activity | MET-min/week |
| Medical History | Coronary Heart Disease | Yes / No (Self-reported diagnosis) |
|  | Stroke | Yes / No (Self-reported diagnosis) |
|  | Hypertension | SBP ≥140 mmHg, DBP ≥90 mmHg, Diagnosis by a physician, Use of antihypertensive medications |
|  | Hyperlipidemia | TG ≥150 mg/dL, TC ≥200 mg/dL, LDL-C ≥130 mg/dL, HDL-C <40 mg/dL, or lipid-lowering medication use |
|  | Diabetes | Physician-diagnosed or lab-based criteria (HbA1c >6.5%, fasting glucose >7.0 mmol/L, random glucose >11.1 mmol/L, or 2-hour glucose tolerance test >11.1 mmol/L) |

**Abbreviations:** PIR, poverty-income ratio; MET, metabolic equivalent of task; SBP, systolic blood pressure; DBP, diastolic blood pressure; TG, triglycerides; TC, total cholesterol; LDL-C, low-density lipoprotein cholesterol; HDL-C, high-density lipoprotein cholesterol; HbA1c, hemoglobin A1c; FPG, fasting plasma glucose.
