# Supplementary material for: Associations between eight anthropometric indices and Parkinson’s disease: a nationwide population-based study
Source: Front Nutr. 2025 Jun 27;12:1621658. doi: 10.3389/fnut.2025.1621658 (PMC12245708; doi:10.3389/fnut.2025.1621658)
Supplement: Supplementary file 7 [file Table_2.doc]

**Supplementary Table 2** The baseline characteristics of anthropometric indicators after Z-score transformation, weighted.

| **Characteristics** | **Participants^a^** | | | |
| --- | --- | --- | --- | --- |
|  | **Overall** | **Non-PD** | **PD** | ***P*-value** |
|  | **n = 41,374** | **n = 41,020** | **n = 354** |  |
| BMI Z- score,(mean (SD)) | -0.029 (0.992) | -0.030 (0.992) | 0.108 (0.991) | **0.0446** |
| WC Z-score ,(mean (SD)) | -0.027 (1.009) | -0.029 (1.009) | 0.272 (0.944) | **<0.0001** |
| WT Z-score,(mean (SD)) | 0.037 (1.005) | 0.036 (1.006) | 0.088 (0.942) | 0.5226 |
| ABSI Z-score, (mean (SD)) | -0.071 (0.965) | -0.075 (0.964) | 0.466 (0.946) | **<0.0001** |
| BRI Z-score, (mean (SD)) | -0.073 (0.986) | -0.076 (0.986) | 0.260 (0.992) | **<0.0001** |
| WWI Z-score, (mean (SD)) | -0.124 (0.970) | -0.128 (0.969) | 0.425 (0.905) | **<0.0001** |
| WHtR Z-score, (mean (SD)) | -0.077 (0.994) | -0.080 (0.994) | 0.274 (0.970) | **<0.0001** |
| CI Z-score, (mean (SD)) | -0.076 (0.990) | -0.080 (0.989) | 0.459 (0.902) | **<0.0001** |

For continuous variables: survey-weighted mean (SD), *P*-value was by survey-weighted linear regression.

**Abbreviations:** BMI, body mass index; WT, weight; WC, waist circumference; WHtR, waist-to-height ratio; BRI, body roundness index; ABSI, A Body Shape Index; WWI, weight-adjusted waist index; CI, conicity index, PD, Parkinson’s disease; SD, standard deviation.
