# Supplementary material for: Associations between eight anthropometric indices and Parkinson’s disease: a nationwide population-based study
Source: Front Nutr. 2025 Jun 27;12:1621658. doi: 10.3389/fnut.2025.1621658 (PMC12245708; doi:10.3389/fnut.2025.1621658)
Supplement: Supplementary file 8 [file Table_3.doc]

**Supplementary Table 3** Associations between anthropometric indices and PD by quartiles.

| **Variables** | **Model 1** | | **Model 2** | | **Model 3** | |
| --- | --- | --- | --- | --- | --- | --- |
|  | **OR (95%CI)** | ***P*-value** | **OR (95%CI)** | ***P*-value** | **OR (95%CI)** | ***P*-value** |
| **WWI(Quartile)** |  |  |  |  |  |  |
| Q1 | 1(Ref) |  | 1(Ref) |  | 1(Ref) |  |
| Q2 | 2.80(1.65, 4.75) | **<0.001** | 2.32(1.37, 3.94) | **0.002** | 2.36(1.36, 4.08) | **0.002** |
| Q3 | 3.89 (2.23, 6.77) | **<0.001** | 2.77(1.58, 4.83) | **<0.001** | 2.71(1.56, 4.72) | **<0.001** |
| Q4 | 6.27 (3.83, 10.28) | **<0.001** | 3.73(2.18, 6.36) | **<0.001** | 3.22(1.77, 5.85) | **<0.001** |
| *P* for trend |  | **<0.001** |  | **<0.001** |  | **<0.001** |
| **CI(Quartile)** |  |  |  |  |  |  |
| Q1 | 1(Ref) |  | 1(Ref) |  | 1(Ref) |  |
| Q2 | 1.81(1.08, 3.02) | **0.024** | 1.57(0.94, 2.62) | 0.085 | 1.55(0.93, 2.59) | 0.089 |
| Q3 | 2.42 (1.42, 4.11) | **0.001** | 1.86(1.08, 3.19) | **0.025** | 1.82(1.02, 3.23) | **0.042** |
| Q4 | 4.6 (2.88, 7.36) | **<0.001** | 3.05(1.87, 5.00) | **<0.001** | 2.74(1.67, 4.49) | **<0.001** |
| *P* for trend |  | **<0.001** |  | **<0.001** |  | **<0.001** |
| **ABSI(Quartile)** |  |  |  |  |  |  |
| Q1 | 1(Ref) |  | 1(Ref) |  | 1(Ref) |  |
| Q2 | 1.85(1.07, 3.19) | **0.028** | 1.72(0.99, 2.98) | 0.053 | 1.67(0.96, 2.90) | 0.068 |
| Q3 | 2.56 (1.61, 4.07) | **<0.001** | 2.10(1.31, 3.36) | **0.002** | 1.96(1.20, 3.18) | **0.007** |
| Q4 | 4.71 (2.88, 7.71) | **<0.001** | 3.08(1.84, 5.16) | **<0.001** | 2.61(1.53, 4.43) | **<0.001** |
| *P* for trend |  | **<0.001** |  | **<0.001** |  | **<0.001** |
| **BRI(Quartile)** |  |  |  |  |  |  |
| Q1 | 1(Ref) |  | 1(Ref) |  | 1(Ref) |  |
| Q2 | 1.71(1.04, 2.79) | **0.033** | 1.35(0.81, 2.24) | 0.243 | 1.40(0.82, 2.42) | 0.219 |
| Q3 | 1.55 (0.90, 2.66) | 0.111 | 1.10(0.63, 1.92) | 0.742 | 1.12(0.64, 1.97) | 0.689 |
| Q4 | 2.75 (1.79, 4.22) | **<0.001** | 1.86(1.19, 2.92) | **0.007** | 1.74(1.05, 2.88) | **0.031** |
| *P* for trend |  | **<0.001** |  | **0.013** |  | **0.039** |
| **WHtR(Quartile)** |  |  |  |  |  |  |
| Q1 | 1(Ref) |  | 1(Ref) |  | 1(Ref) |  |
| Q2 | 1.71(1.04, 2.79) | **0.033** | 1.35(0.81, 2.24) | 0.243 | 1.40(0.82, 2.42) | 0.219 |
| Q3 | 1.55(0.90, 2.66) | 0.111 | 1.10(0.63, 1.92) | 0.742 | 1.12(0.64, 1.97) | 0.689 |
| Q4 | 2.75 (1.79, 4.22) | **<0.001** | 1.86(1.19, 2.92) | **0.007** | 1.74(1.05, 2.88) | **0.031** |
| *P* for trend |  | **<0.001** |  | **0.013** |  | **0.039** |
| **BMI (Quartile)** |  |  |  |  |  |  |
| Q1 | 1(Ref) |  | 1(Ref) |  | 1(Ref) |  |
| Q2 | 0.82(0.51, 1.34) | 0.429 | 0.75(0.46, 1.22) | 0.249 | 0.80(0.49, 1.28) | 0.347 |
| Q3 | 1.10 (0.68, 1.78) | 0.686 | 0.98(0.62, 1.56) | 0.939 | 1.02 (0.66,1.57) | 0.935 |
| Q4 | 1.26 (0.86, 1.85) | 0.238 | 1.19(0.81, 1.74) | 0.380 | 1.15 0.79, 1.69) | 0.463 |
| *P* for trend |  | 0.145 |  | 0.243 |  | 0.29 |
| **WC (Quartile)** |  |  |  |  |  |  |
| Q1 | 1(Ref) |  | 1(Ref) |  | 1(Ref) |  |
| Q2 | 1.37(0.81, 2.31) | 0.240 | 1.200.70, 2.06) | 0.495 | 1.25(0.71, 2.19) | 0.436 |
| Q3 | 1.58 (0.98, 2.55) | 0.058 | 1.30(0.80, 2.10) | 0.291 | 1.33 (0.80,2.23) | 0.273 |
| Q4 | 2.54 (1.61, 3.99) | **<0.001** | 2.13(1.36, 3.34) | **0.001** | 2.10(1.31, 3.37) | **0.002** |
| *P* for trend |  | **<0.001** |  | **<0.001** |  | **<0.001** |
| **WT (Quartile)** |  |  |  |  |  |  |
| Q1 | 1(Ref) |  | 1(Ref) |  | 1(Ref) |  |
| Q2 | 0.91(0.59, 1.40) | 0.671 | 0.99(0.65, 1.52) | 0.977 | 1.03(0.68, 1.55) | 0.895 |
| Q3 | 1.29 (0.81, 2.05) | 0.276 | 1.53(0.97, 2.42) | 0.068 | 1.61(1.04, 2.51) | **0.034** |
| Q4 | 1.26 (0.78, 2.04) | 0.344 | 1.66(1.04, 2.67) | **0.036** | 1.74(1.14, 2.66) | **0.011** |
| *P* for trend |  | 0.175 |  | **0.01** |  | **0.002** |

***Abbreviations:*** OR, odds ratio; CI, confidence interval; PD, Parkinson’s disease; WWI, weight-adjusted waist index; CI, conicity index; ABSI, a body shape index; BRI, body roundness index; WHtR, waist-to-height ratio;BMI, body mass index; WC, waist circumference; WT, weight.
